# The “telomereless” erythrocytes and telomere‐length dependent erythropoiesis

**DOI:** 10.1111/acel.13997

**Published:** 2023-10-12

**Authors:** Abraham Aviv

**Affiliations:** ^1^ Center of Human Development and Aging New Jersey Medical School, Rutgers The State University of New Jersey Newark New Jersey USA

**Keywords:** aging, anemia, biomarker, erythropoiesis, hematopoiesis, leukocytes

## Abstract

Approximately 25 trillion erythrocytes (red blood cells) circulate in the bloodstream of an adult human, surpassing the number of circulating leukocytes (white blood cells) by a factor of about 1000. Moreover, the erythrocyte turnover rate accounts for approximately 76% of the turnover rate of all circulating blood cells. This simple math shows that the hematopoietic system principally spends its telomere length‐dependent replicative capacity on building and maintaining the erythrocyte blood pool. Erythropoiesis (red blood cell production) is thus the principal cause of telomere shortening with age in hematopoietic cells (HCs), a conclusion that holds significant implications for linking telomere length dynamics in HCs to health and lifespan of modern humans.

## Graphical abstract

The hematopoietic system primarily allocates its telomere length‐dependent replicative capability toward building and sustaining the population of erythrocytes (red blood cells) in the circulation. Therefore, telomere shortening with age in hematopoietic cells is primarily driven by erythropoiesis (red blood cell production).

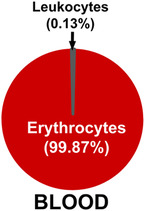

**Abbreviations**
BMF: bone marrow failure
bp: base pair
HCs: hematopoietic cells
HSC: hematopoietic stem cell
kb: kilobase
LTL: leukocyte telomere length
mTL: mean telomere length
TBDs: telomere biology disorders
TL: telomere length

## 1 INTRODUCTION

*Omnis cellua e cellula*, “All cells come from cells.” Attributed to Virchow, this cell‐replication maxim originates from an 1825 manuscript by Raspail (Wright & Poulsom, 2012). Three scientific milestones that occurred about one and a half centuries later ushered in the golden era of fundamental telomere research, which continues today. First, Hayflick & Moorhead (1961) showed that cultured human fibroblasts experience a finite number of replications, ultimately reaching a state now known as replicative senescence (Shay & Wright, 2000). Second, Olovnikov (1973) and Watson (1972) independently predicted the “end‐replication problem” whereby telomeres progressively shorten during cell replication because DNA polymerase is unable to complete the replication of 3′ end of the telomeres. Third, Greider & Blackburn (1985) discovered telomerase, the reverse transcriptase that elongates telomeres. These developments also promoted population‐based telomere studies.

Telomere length (TL) displays robust correlations between somatic cells within a person (Daniali et al., 2013; Kimura et al., 2010) but vast variation across individuals (Aubert et al., 2012; Daniali et al., 2013; Factor‐Litvak et al., 2016; Steenstrup et al., 2017). Researchers have thus used TL of leukocytes (white blood cells) as a proxy measure for TL in all somatic cells. However, TL of somatic cells reflects their replication history such that cells that underwent more replications have shorter telomeres than those that underwent fewer replications. For instance, in adults, leukocyte TL (LTL), which represents the highly proliferative hematopoietic system, is shorter by about 1400 base pairs (bp) than TL of skeletal muscle, a minimally proliferative tissue (Daniali et al., 2013).

Yet, as discussed here, the hematopoietic system ostensibly spends most of its TL‐dependent replicative capacity not on producing leukocytes but on making the enucleated and thus “telomereless” erythrocytes (red blood cells). This raises a semantic matter: The focus of population studies on LTL has prompted the idea that leukocyte turnover largely explains age‐dependent LTL shortening. Hematopoietic cell (HC) TL, however, is a better term to describe TL dynamics (TL and its shortening with age) throughout the hematopoietic hierarchy. For clarity, I retain the term LTL when referring to published findings.

## 2 VIEWS ON HUMAN LTL

Two major views govern population‐based telomere research (Aviv & Shay, 2018). A widely held view regards LTL as a biological timekeeper, that is, a biomarker, of human aging. It suggests that an adult whose LTL ranks short on the LTL distribution in the general population has experienced a faster leukocyte telomere shortening, and hence a faster biological aging, than another adult of the same age whose LTL ranks long on the distribution. Such thinking became entwined with the notion that environmental factors and unhealthy lifestyle accelerate the age‐dependent shortening of leukocyte telomeres (Bountziouka et al., 2022; Pepper et al., 2018). It was further perpetuated by the widespread reliance on a quantitative (q)PCR‐ based method that estimates TL as the amplified telomere product (T) scaled to a single‐gene product (S) (Cawthon, 2009). T/S data provide no awareness of the “biological numeracy” (Phillips & Milo, 2009) of TL, as expressed, for instance, in its variation in absolute TL units within and across individuals, and the rate of its shortening with age.

The weight of evidence now supports a second view: Having long or short LTL hardly reflects leukocyte telomere shortening with age but instead is a function of interindividual differences in LTL at birth. That is because LTL variation among newborns (Factor‐Litvak et al., 2016), standard deviation of ~700 bp, which is largely determined by heredity (Hjelmborg et al., 2015), equals that observed in adults (Factor‐Litvak et al., 2016; Steenstrup et al., 2017). Such variation means that among newborns the telomere timekeeper does not start at “biological time” zero but rather at varied biological times. By such logic, newborns whose LTL ranks low on the LTL distribution are biologically older than newborns whose LTL ranks high. This would imply that many individuals are biologically older right from birth. Moreover, from the second decade onwards, LTL shows stable “tracking” such that individuals retain their relative ranking in the LTL distribution, that is, having longer or shorter LTL, as they age (Benetos et al., 2013; Benetos et al., 2019). Jointly, these findings indicate that in most individuals, the interindividual differences in the rate of leukocyte telomere shortening after birth do not offset the LTL variation that has been established prior to adulthood. Birth LTL is thus the main determinant of LTL ranking throughout the life course.

Such findings also suggest that LTL contributes causally to many LTL‐associated diseases, because LTL ranking predates by decades the onset of these diseases. Genetically determined LTL, based on LTL‐associated single nucleotide polymorphisms, and Mendelian randomization analyses, supports this conjecture (Codd et al., 2021; Haycock et al., 2014). A broad question then arises: What drives leukocyte telomere shortening with age? The answer requires knowledge about how hematopoiesis (blood cell production) shortens HC telomeres.

## 3 HC TELOMERE SHORTENING DURING HEMATOPOIESIS

**FIGURE 1:**
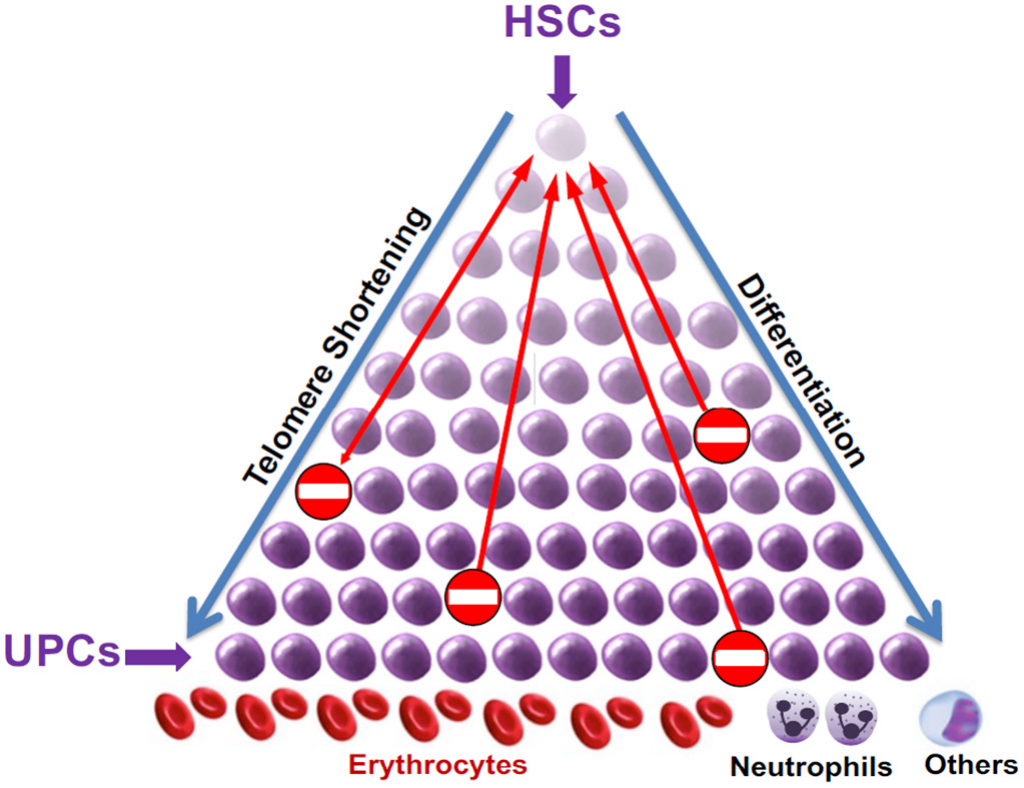
The hematopoietic hierarchy. Hematopoietic stem cells (HSCs) are at the top of the hierarchy and unipotent progenitor cells (UPCs) are at the bottom. No entry sign denotes hematopoietic cells that have reached replicative senescence, prompting signaling for increased replication of cells up the hierarchy.

The 50,000–200,000 long‐lived hematopoietic stem cells atop the hematopoietic hierarchy of an adult (Lee‐Six et al., 2018) replicate about once a year, yet they give rise to hundreds of billions of new blood cells released daily from the bone marrow into the circulation (Boyle et al., 2023; Dingli et al., 2007; Shepherd et al., 2004; Werner et al., 2015). This massive output of blood cells is the outcome of many sequential HC replications that follow the initial replication of a hematopoietic stem cell. These replications happen for approximately 90 days across the hierarchy, until the ultimate progenies of a hematopoietic stem cell at the apex are released into the circulation (Dingli et al., 2007; Werner et al., 2015). During this time, while the HCs differentiate from multipotent to oligopotent and then short‐lived unipotent progenitor cells, their replicative pace progressively increases down the hierarchy and their numbers rise exponentially (Figure 1).

The nominal activity of telomerase in HCs (Lansdorp, 2022; Zimmermann & Martens, 2008) is insufficient to prevent replication‐mediated TL shortening, which might ultimately trigger replicative senescence. Moreover, the hierarchal nature of hematopoiesis means that TL‐dependent replicative senescence of HCs at any level of the hierarchy generates demands that ripple upward for more replication of cells up the hierarchy to replenish HCs that depleted their replicative capacity at the bottom (Figure 1). Increased hematopoietic stem cell replication at the top of the hematopoietic hierarchy thus causes TL shortening with age in all HC lineages.

These lineages include myeloid and lymphoid cells. The myeloid lineage primarily includes erythrocytes and neutrophils (type of white blood cells), while the lymphoid lineage primarily consists of T cells and B cells. During the final step of erythropoiesis (production of erythrocytes) in the bone marrow, unipotent progenitor cells called erythroblasts lose their nuclei to become erythrocytes (Moras et al., 2017). Lacking nuclei, erythrocytes cannot replicate in the circulation. Though having nuclei, neutrophils are terminally differentiated, and they too do not replicate in the circulation. Lymphocytes continue to replicate and differentiate in extramedullary sites, explaining their shorter telomeres compared to neutrophils' telomeres in adults (Aubert et al., 2012).

## 4 THE ENUCLEATED ERYTHROCYTES ARE THE DOMINANT CELLS IN THE SOMA AND BLOOD

**FIGURE 2:**
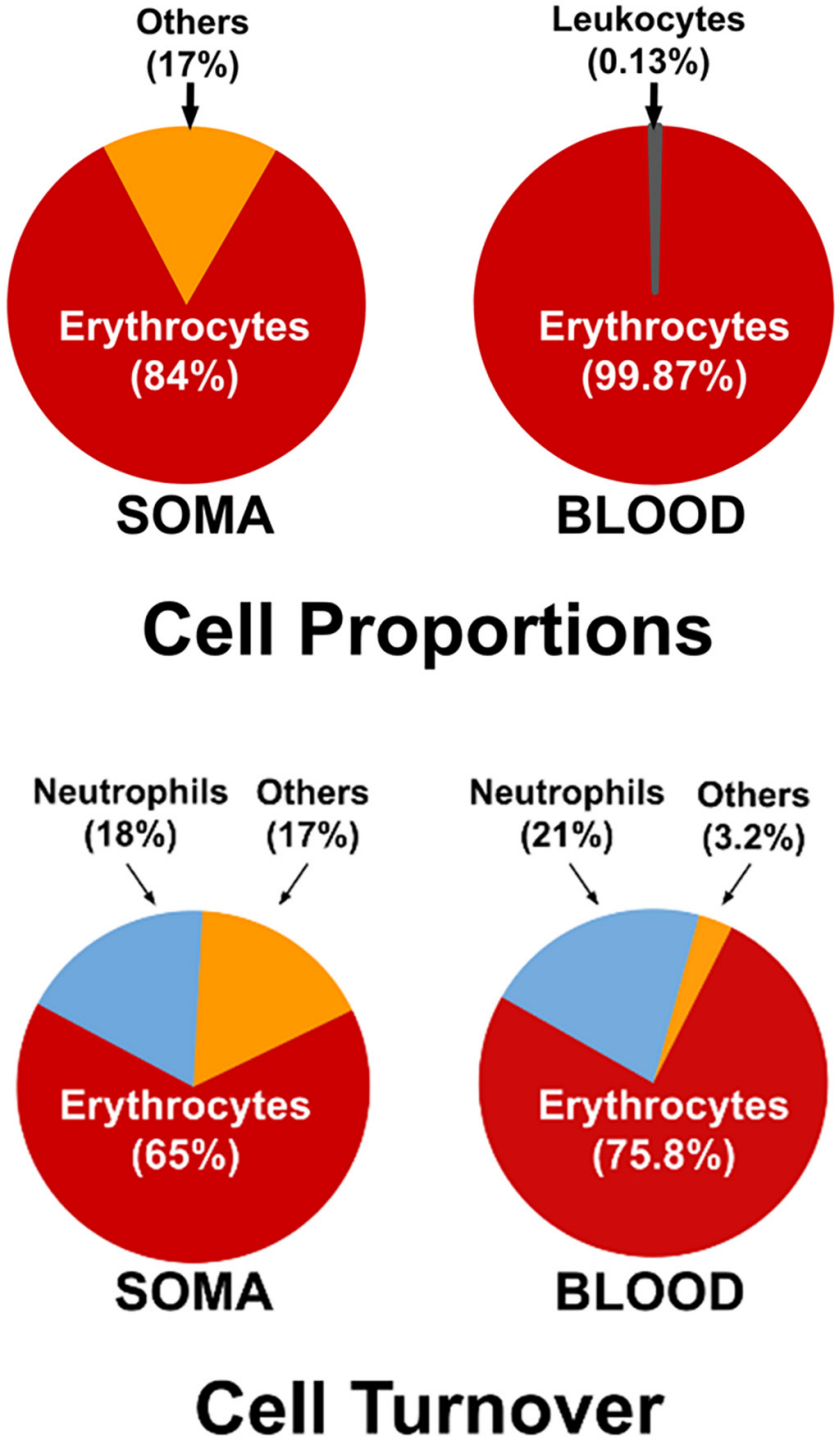
Cell proportions and daily cellular turnovers in the soma and blood of a normal adult. The top panel draws on data from reference Sender et al., 2016 (left), and complete blood cell count with differential (https://www.bowtie.com.hk/blog/en/full‐blood‐count/) (right). Values in the bottom panel are expressed as the percentage of total somatic cell turnover, drawing on data from reference Sender & Milo, 2021 (left), and data rescaled from somatic cell turnovers (right).

While researchers typically measure TL in circulating leukocytes, principally neutrophils and lymphocytes, erythrocytes are the prevailing blood cells. The 25 trillion erythrocytes in an average adult comprise 84% of the 30 trillion somatic cells and 99.87% of circulating blood cells (Figure 2) (Sender et al., 2016). These numbers indicate that a prime portion of TL‐dependent replicative capacity of the hematopoietic system and the soma in general is used for erythropoiesis. However, a “dynamic” model of somatic cell production, which reflects cell turnover rate, provides a better account of demands imposed on the TL‐dependent replicative capacity than the “static” picture of the number of somatic cells.

## 5 THE DAILY TURNOVER OF ERYTHROCYTES EXCEEDS THE COMBINED TURNOVER OF ALL SOMATIC CELLS

Figure 2 also displays the daily turnover of somatic cells, defined as the absolute number of a specific cell type divided by its lifespan (in days) under steady state (Sender & Milo, 2021). The lifespans of somatic cells, ranging from hours to years, exert a strong effect on their turnover. Because of their sheer magnitude in numbers, despite their 120‐day lifespan, the turnover of erythrocytes accounts for 65% of the turnover of all somatic cells combined. The daily turnover of neutrophils reflects their presence not only in the circulation but also in the bone marrow. Updated data suggest that neutrophil lifespan is about 22 h in the circulation and 5.7 days in the bone marrow (Sender & Milo, 2021). This time places the turnover of neutrophils at 18% of all somatic cells, second to that of erythrocytes. Together, the turnovers of erythrocytes and neutrophils amounts to 83% of the turnover of all somatic cells. Among circulating blood cells, the respective turnovers of erythrocytes and neutrophils are 75.8% and 21%, jointly amounting to 96.8% of the turnover of all blood cells.

## 6 ANEMIA AND TL‐DEPENDENT REPLICATIVE CAPACITY OF HCS

If making an erythrocyte and a neutrophil requires the same amount of TL‐dependent HC replicative capacity, about three fourth and one fifth of this capacity are spent in an average adult on producing erythrocytes and neutrophils, respectively. These numbers do not consider the massive amount of TL invested in building the erythrocyte blood poll during growth and development. Lymphocytes are also produced through clonal expansion out of the bone marrow, but as the overall lymphocyte lifespan is long (months to years) (Vrisekoop et al., 2008), lymphocyte turnover exerts a minimal effect on TL‐dependent replicative rate of bone marrow HCs.

Accordingly, anemia might be a cardinal sign in individuals whose HC TL is too short to maintain daily erythropoiesis. This is shown by rare diseases known collectively as telomere biology disorders (TBDs) (Townsley et al., 2014). LTL below the first percentile of its distribution in the general population is a defining feature of TBDs, which stem from detrimental mutations in telomere maintenance genes. Patients might present with multisystem findings, but often suffer from bone marrow failure (BMF), expressed in aplastic anemia and neutropenia. The development of aplastic anemia in TBDs provides evidence that critically short telomeres can curtail replication of human somatic cells in vivo (Raj et al., 2023). To some extent, mice with genetically engineered deficiencies in telomere‐maintenance genes recapitulate hematological findings observed in patients with TBDs (Calado & Dumitriu, 2013). However, key differences in TL‐dependent BMF distinguish humans from mice. Namely, wild type mice live for about 2 years and most mice have long telomeres, that is, mean telomere length (mTL) ~ 30 kb (Calado & Dumitriu, 2013; Gomes et al., 2011). Sustained deficiency in telomere maintenance across several generations of genetically engineered mice is thus necessary to shorten their telomeres to a length that causes aplastic anemia. Humans, the longest‐living terrestrial mammals, have very short telomeres, that is, mTL ~ 9.5 kb at birth (Factor‐Litvak et al., 2016), and in some patients with severe forms of TBDs, BMF may develop during early childhood.

## 7 SUMMARY

Building the massive erythrocyte pool during childhood and maintaining it throughout life are the principal causes of LTL shortening after birth. The aging‐biomarker view has overlooked these dynamics. It has prompted numerous cross‐sectional studies that examined the influence of environmental exposures and human behaviors on LTL. Performed principally in adults, these studies aimed to show that environments and behaviors alter the pace of age‐dependent LTL shortening, and that by slowing LTL shortening, healthy lifestyle diminishes risk for aging‐related diseases. However, meta‐analysis of 138 of these studies, and an interrogation of UK Biobank data, which jointly comprise about 800,000 persons, found no credible evidence that exposures and behaviors during adulthood influence LTL at a magnitude that might be consequential for human health and longevity (Bountziouka et al., 2022; Pepper et al., 2018). These findings are consistent with the view that in middle‐high‐income societies LTL ranking is principally determined at birth (Benetos et al., 2013; Benetos et al., 2019; Factor‐Litvak et al., 2016). It's still unknown, however, whether environmental factors in these societies might significantly affect LTL ranking in utero and early childhood.

Finally, under normal circumstances, in most individuals, HCs appear to have adequate TL‐dependent replicative reserves throughout the present human lifespan, avoiding a drastic decrease in the number of circulating erythrocytes as shown in patients with TBDs (Lai et al., 2023). The inferred causal role of LTL in a host of human diseases (Codd et al., 2021; Haycock et al., 2014) may arise from other mechanisms, including different forms of clonal expansions (Anderson et al., 2022; Aviv et al., 2017; Nakao et al., 2022). It is important to note, however, that approximately 10% of individuals over the age of 65 and more than 20% of those over 85, suffer from anemia. What is intriguing is that in one third of these cases, the cause of anemia remains unexplained (Guralnik et al., 2004). As LTL correlates with the number of circulating erythrocytes (Codd et al., 2021) and the lifespan of mammals correlates with the rate of erythropoiesis (Udroiu & Sgura, 2019), it is possible that the unexplained anemia in these elderly individuals is partially attributable to a decline in TL‐dependent erythropoiesis.

## 8 THE WAY FORWARD

Though gene–environment interactions drive mammalian evolution (Snyder‐Mackler & Lea, 2018), environments now affecting residents of Boston and London differ drastically from those that molded TL‐linked erythropoiesis in archaic humans. That is why population‐based telomere research will benefit from longitudinal studies of HC TL dynamics starting at birth in peoples of diverse ancestries and geographies. New telomere data from these peoples will forge novel insights into the role of telomeres in building the hematopoietic system and the role of HC TL in health and longevity of contemporary humans.

## 9 AUTHOR CONTRIBUTIONS

Dr. Aviv is the sole author for this manuscript. He is responsible for the conception of the paper, acquisition and interpretation of data from published papers.

## FUNDING INFORMATION

The author's telomere research is supported by National Institutes of Health grants R01 HL134840, U01AG066529,1R56AG073226, and a grant from the Norwegian Research Council (NFR) ES562296.

## CONFLICT OF INTEREST STATEMENT

The author declares no competing financial interest.

## Data Availability

Data sharing is not applicable to this article as no new data were created or analyzed in this study.
